# Supplementary material for: End Sequence Analysis Toolkit (ESAT) expands the extractable information from single-cell RNA-seq data
Source: Genome Res. 2016 Oct;26(10):1397–410. doi: 10.1101/gr.207902.116 (PMC5052061; doi:10.1101/gr.207902.116)
Supplement: Supplemental Material [file supp_gr.207902.116_Supp_Materials.tar.gz › ESAT-esat_v0.1/doc/html/classbroad_1_1pda_1_1annotation_1_1_g_t_f_file_parser-members.html]

ESAT: Member List


ESAT


- Main Page
- Related Pages
- Packages
- Classes
- Files

- Class List
- Class Index
- Class Hierarchy
- Class Members

All Classes Namespaces Files Functions Variables Enumerator Pages

- broad
- pda
- annotation
- GTFFileParser

broad.pda.annotation.GTFFileParser Member List

This is the complete list of members for broad.pda.annotation.GTFFileParser, including all inherited members.

|  |  |  |
| --- | --- | --- |
| addRefSeq(Gene gene) | broad.pda.annotation.BEDFileParser |  |
| addRefSeq(GeneWithIsoforms gene) | broad.pda.annotation.BEDFileParser |  |
| addRefSeqSet(Collection<?extends Gene > collection) | broad.pda.annotation.BEDFileParser |  |
| bed2CuffGtf(BufferedWriter bw, String src, boolean useExtraFileds) | broad.pda.annotation.BEDFileParser |  |
| bed2gtf(BufferedWriter bw, String src) | broad.pda.annotation.BEDFileParser |  |
| bed2gtf\_bundleOverlappers(BufferedWriter bw, String src, BEDFileParser mergedData) | broad.pda.annotation.BEDFileParser |  |
| BEDFileParser() | broad.pda.annotation.BEDFileParser |  |
| BEDFileParser(String fileName) | broad.pda.annotation.BEDFileParser |  |
| BEDFileParser(String fileName, int ThreePrimeTrimSize) | broad.pda.annotation.BEDFileParser |  |
| BEDFileParser(String fileName, String chr) | broad.pda.annotation.BEDFileParser |  |
| BEDFileParser(Collection< Gene > genes) | broad.pda.annotation.BEDFileParser |  |
| BEDFileParser(Map< String, Collection< Gene >> transcripts) | broad.pda.annotation.BEDFileParser |  |
| clear() | broad.pda.annotation.BEDFileParser |  |
| collapseSingleExonTranscripts() | broad.pda.annotation.BEDFileParser |  |
| containChr(String chr) | broad.pda.annotation.BEDFileParser |  |
| contains(String geneName) | broad.pda.annotation.BEDFileParser |  |
| copy() | broad.pda.annotation.BEDFileParser |  |
| dedup() | broad.pda.annotation.BEDFileParser |  |
| differenceByGenomicRegion(BEDFileParser other, String considerOrientaion) | broad.pda.annotation.BEDFileParser |  |
| equalizeTranscriptEnds(BEDFileParser other) | broad.pda.annotation.BEDFileParser |  |
| expanedUtrs(BEDFileParser bed, Integer utr1, Integer utr2) | broad.pda.annotation.BEDFileParser | static |
| filterByOverlap(Collection< Gene > genes) | broad.pda.annotation.BEDFileParser |  |
| get(String geneName) | broad.pda.annotation.BEDFileParser |  |
| get3PRegion(int toIn, int toOut) | broad.pda.annotation.BEDFileParser |  |
| getAnnotationSetMap() | broad.pda.annotation.BEDFileParser |  |
| getChromosomeIterator() | broad.pda.annotation.BEDFileParser |  |
| getChrTree(String chr) | broad.pda.annotation.BEDFileParser |  |
| getClosestDownstream(Gene annot) | broad.pda.annotation.BEDFileParser |  |
| getClosestDownstream(LightweightGenomicAnnotation annot) | broad.pda.annotation.BEDFileParser |  |
| getClosestUpstream(Gene annot) | broad.pda.annotation.BEDFileParser |  |
| getClosestUpstream(LightweightGenomicAnnotation annot) | broad.pda.annotation.BEDFileParser |  |
| getExactIsoform(GeneWithIsoforms loci) | broad.pda.annotation.BEDFileParser |  |
| getGene(String geneFile, String geneName) | broad.pda.annotation.BEDFileParser | static |
| getGeneByName(String geneName, String bedFile) | broad.pda.annotation.BEDFileParser | static |
| getGeneIDIsoMap() | broad.pda.annotation.GTFFileParser |  |
| GetGenes() | broad.pda.annotation.BEDFileParser |  |
| GetGenesWithIsoforms() | broad.pda.annotation.BEDFileParser |  |
| getGenomicRegionOverlappers(LightweightGenomicAnnotation region) | broad.pda.annotation.BEDFileParser |  |
| getGTFGeneTranscriptMap() | broad.pda.annotation.GTFFileParser |  |
| getIntervalTreeWithIsoforoms() | broad.pda.annotation.BEDFileParser |  |
| getMergedAnnotationMap(double minPctOverlapToMerge) | broad.pda.annotation.BEDFileParser |  |
| getMergedAnnotationMap(boolean mergall) | broad.pda.annotation.BEDFileParser |  |
| getMergedCopy() | broad.pda.annotation.BEDFileParser |  |
| getNameGeneMap() | broad.pda.annotation.BEDFileParser |  |
| getNumberOfIsoforms(String chr) | broad.pda.annotation.BEDFileParser |  |
| getNumberOfIsoforms() | broad.pda.annotation.BEDFileParser |  |
| getNumberOfIsoforms(IntervalTree< GeneWithIsoforms > overlapTree) | broad.pda.annotation.BEDFileParser | static |
| getOverlappers(Gene gene) | broad.pda.annotation.BEDFileParser |  |
| getOverlappers(LightweightGenomicAnnotation region) | broad.pda.annotation.BEDFileParser |  |
| getPromoters() | broad.pda.annotation.BEDFileParser |  |
| getPromoters(int toIn, int toOut) | broad.pda.annotation.BEDFileParser |  |
| GTFFileParser() | broad.pda.annotation.GTFFileParser |  |
| GTFFileParser(String fileName) | broad.pda.annotation.GTFFileParser |  |
| GTFFileParser(String fileName, String chr) | broad.pda.annotation.GTFFileParser |  |
| GTFWrite(BufferedWriter bw, String Src, boolean UseExtraFields) | broad.pda.annotation.GTFFileParser |  |
| IncrementScoreIfOverlap(BEDFileParser bed, int setNumber, Boolean setToZero) | broad.pda.annotation.BEDFileParser |  |
| intersect(BEDFileParser bed) | broad.pda.annotation.BEDFileParser |  |
| isEmpty() | broad.pda.annotation.BEDFileParser |  |
| isOverlapCompatible(Gene first, Gene second, double minPctOverlap) | broad.pda.annotation.BEDFileParser | static |
| loadAlignmentData(File file) | broad.pda.annotation.BEDFileParser | static |
| loadAlignmentData(BufferedReader reader) | broad.pda.annotation.BEDFileParser | static |
| loadAlignmentData(File file, boolean ucscDownload) | broad.pda.annotation.BEDFileParser | static |
| loadAlignmentDataByChr(File file) | broad.pda.annotation.BEDFileParser | static |
| loadAlignmentDataByChr3Prime(File file, int extensionFactor) | broad.pda.annotation.BEDFileParser | static |
| loadAlignmentDataByChr5Prime(File file, int extensionFactor) | broad.pda.annotation.BEDFileParser | static |
| loadAlignmentDataByChrArrayList(File file) | broad.pda.annotation.BEDFileParser | static |
| loadAlignmentDataToTree(File file) | broad.pda.annotation.BEDFileParser | static |
| loadChrSizes(String file) | broad.pda.annotation.BEDFileParser | static |
| loadData(File file) | broad.pda.annotation.BEDFileParser | static |
| loadData(File file, Alignments region) | broad.pda.annotation.BEDFileParser | static |
| loadDataByChr(String file) | broad.pda.annotation.BEDFileParser | static |
| loadDataByChr(File file) | broad.pda.annotation.BEDFileParser | static |
| loadDataByChrToTree(File file) | broad.pda.annotation.BEDFileParser | static |
| loadDataByName(File file) | broad.pda.annotation.BEDFileParser | static |
| loadDataLine(File file) | broad.pda.annotation.BEDFileParser | static |
| loadDataLine(BufferedReader reader) | broad.pda.annotation.BEDFileParser | static |
| loadGTF(File file, String chr) | broad.pda.annotation.BEDFileParser |  |
| loadIsoformDataByChrToTree(File file, String chr) | broad.pda.annotation.GTFFileParser | protected |
| loadIsoformDataByChrToTree(File file) | broad.pda.annotation.GTFFileParser | protected |
| loadList(String file) | broad.pda.annotation.BEDFileParser | static |
| loadList(String file, boolean skipFirstLine) | broad.pda.annotation.BEDFileParser | static |
| main(String[] args) | broad.pda.annotation.BEDFileParser | static |
| makeGenes() | broad.pda.annotation.BEDFileParser |  |
| makeGenes(double minPctOverlapToMerge) | broad.pda.annotation.BEDFileParser |  |
| makeIntervalTreeFor3Prime(File geneFile, int size) | broad.pda.annotation.BEDFileParser | static |
| merge() | broad.pda.annotation.BEDFileParser |  |
| mergeByGtfGeneId() | broad.pda.annotation.BEDFileParser |  |
| minus(Collection< Gene > genes, boolean recursive) | broad.pda.annotation.BEDFileParser |  |
| overlap\_GenomicLevel(BEDFileParser other, String task, String considerOrientaion) | broad.pda.annotation.BEDFileParser |  |
| overlapByGenomicRegion(BEDFileParser other, String considerOrientaion) | broad.pda.annotation.BEDFileParser |  |
| readSiphyOutToBed(String siphyFile) | broad.pda.annotation.BEDFileParser | static |
| remove(GeneWithIsoforms record) | broad.pda.annotation.BEDFileParser |  |
| removeSingeleExon() | broad.pda.annotation.BEDFileParser |  |
| removeSmallTranscripts(int minExnNum, int minTranscriptLength) | broad.pda.annotation.BEDFileParser |  |
| toConstituentIntroformMap() | broad.pda.annotation.BEDFileParser |  |
| toConstituentIsoformMap() | broad.pda.annotation.BEDFileParser |  |
| toConstituentIsoformMap(boolean removeGenesWithoutConstituentIsoforms) | broad.pda.annotation.BEDFileParser |  |
| toMap() | broad.pda.annotation.BEDFileParser |  |
| updateScrToBedScore(double score) | broad.pda.annotation.BEDFileParser |  |
| uploadSetOfFiles(String bedLst) | broad.pda.annotation.BEDFileParser | static |
| write(BufferedWriter bw, String source) | broad.pda.annotation.GTFFileParser |  |
| writeBED(String save, Collection< Annotation > regions) | broad.pda.annotation.BEDFileParser | static |
| writeBED(String save, Map< Annotation, double[]> scores) | broad.pda.annotation.BEDFileParser | static |
| writeBED(String save, Collection< Annotation > alignments, boolean sort) | broad.pda.annotation.BEDFileParser | static |
| writeBED(String save, Map< Annotation, double[]> scores, boolean withScores, boolean sort) | broad.pda.annotation.BEDFileParser | static |
| writeBEDWithScores(String save, Map< Annotation, double[]> scores) | broad.pda.annotation.BEDFileParser | static |
| writeFullBED(String save, Collection< Gene > genes) | broad.pda.annotation.BEDFileParser | static |
| writeFullBED(String save, Map< Gene,?extends Object > genes) | broad.pda.annotation.BEDFileParser | static |
| writeFullBed(String save) | broad.pda.annotation.BEDFileParser |  |
| writeFullBed(String save, boolean useExtraFields) | broad.pda.annotation.BEDFileParser |  |
| writeFullBed(BufferedWriter bw) | broad.pda.annotation.BEDFileParser |  |
| writeFullBed(BufferedWriter bw, boolean useExtraFields) | broad.pda.annotation.BEDFileParser |  |
| writeSortedBED(String save, Map< Annotation, double[]> scores) | broad.pda.annotation.BEDFileParser | static |
| writeSortedBED(String save, Collection< Annotation > alignments) | broad.pda.annotation.BEDFileParser | static |
| writeSortedBEDWithScores(String save, Map< Annotation, double[]> scores) | broad.pda.annotation.BEDFileParser | static |


---

Generated on Fri Aug 15 2014 15:10:58 for ESAT by  

 1.8.7
